# Comparative effectiveness of a portion-controlled meal replacement program for weight loss in adults with and without diabetes/high blood sugar

**DOI:** 10.1038/nutd.2017.32

**Published:** 2017-07-10

**Authors:** C D Coleman, J R Kiel, A H Mitola, L M Arterburn

**Affiliations:** 1 Department of Scientific and Clinical Affairs, Medifast, Inc. 11445 Cronhill Drive, Owings Mills, MD, USA; 2 Independent Consultant, Clifton Park, NY, USA

## Abstract

**Background:**

Individuals with type 2 diabetes (DM2) may be less successful at achieving therapeutic weight loss than their counterparts without diabetes. This study compares weight loss in a cohort of adults with DM2 or high blood sugar (D/HBS) to a cohort of adults without D/HBS. All were overweight/obese and following a reduced or low-calorie commercial weight-loss program incorporating meal replacements (MRs) and one-on-one behavioral support.

**Subjects/Methods:**

Demographic, weight, body composition, anthropometric, pulse and blood pressure data were collected as part of systematic retrospective chart review studies. Differences between cohorts by D/HBS status were analyzed using Mann–Whitney *U*-tests and mixed model regression.

**Results:**

A total of 816 charts were included (125 with self-reported D/HBS). The cohort with D/HBS had more males (40.8 vs 25.6%), higher BMI (39.0 vs 36.3 kg m^−^^2^) and was older (56 vs 48 years). Among clients continuing on program, the cohorts with and without D/HBS lost, on average, 5.6 vs 5.8 kg (NS) (5.0 vs 5.6% *P*=0.005) of baseline weight at 4 weeks, 11.0 vs 11.6 kg (NS) (9.9 vs 11.1% *P*=0.027) at 12 weeks and 16.3 vs 17.1 kg (13.9 vs 15.7% NS) at 24 weeks, respectively. In a mixed model regression controlling for baseline weight, gender and meal plan, and an intention-to-treat analysis, there was no significant difference in weight loss between the cohorts at any time point. Over 70% in both cohorts lost ⩾5% of their baseline weight by the final visit on their originally assigned meal plan. Both cohorts had significant reductions from baseline in body fat, blood pressure, pulse and abdominal circumference.

**Conclusion:**

Adults who were overweight/obese and with D/HBS following a commercial weight-loss program incorporating MRs and one-on-one behavioral support achieved therapeutic weight loss. The program was equally effective for weight loss and reductions in cardiometabolic risk factors among adults with and without D/HBS.

## Introduction

Across the world, ~9% of adults now have diabetes and 7% have prediabetes;^1^ these numbers are expected to rise, largely owing to the growing prevalence of obesity.^2^ Rates are higher in the United States where over 12% of adults have diabetes and another 37% have prediabetes.^3^ Diabetes is now the third leading cause of death, and as many as one in three adults may have diabetes by 2050, making it among the most pressing public health concerns in America.^4, 5^

Being overweight or obese is a major risk factor for developing type 2 diabetes (DM2), and weight loss is a recommended treatment strategy for the prevention and management of this condition. Research has shown that those with prediabetes can reduce their risk of developing DM2 by ~60–80% by weight loss accomplished through participation in a structured lifestyle change program with or without pharmacotherapy.^6, 7^ In people with DM2, modest weight loss of just 2 to <5% can improve glycemic control, and therapeutic weight loss of ⩾5% can further improve glycemia and produce cardiometabolic benefits.^8, 9^ Weight loss of ⩾10% in DM2 has been associated with a reduced rate of cardiovascular events and deaths.^10^

Individuals with DM2 may have a more difficult time losing weight due to metabolic, psychological or behavioral factors, and consequently may be less likely to achieve a therapeutic level of weight loss compared with their peers without diabetes.^11, 12, 13^ However, only a handful of studies have directly compared the weight loss results of individuals with DM2 to those without DM2. Results of those that did were mixed; some studies showed similar weight loss among individuals with and without DM2,^14, 15^ whereas others found people with DM2 tended to lose less weight.^9, 11, 16, 17, 18^ Disease progression and use of some DM2 medications may be a factor in success.^9, 12^

The use of meal replacements (MRs) for weight loss has been shown to be effective in individuals with and without DM2.^9, 14, 15, 19, 20, 21, 22, 23^ MRs provide portion control and can bring structure to weight-loss interventions. Weight-loss success can also be enhanced by providing behavioral support to improve adherence to a reduced calorie diet, using either in-person or telephone counseling.^22, 24, 25^ Including both MRs and support provides a more comprehensive approach to weight reduction and is associated with weight loss success.^26^ Accordingly, recent medical treatment guidelines for the management of overweight and obesity in adults recommend participation in a comprehensive lifestyle program.^27^ Commercial programs with a comprehensive lifestyle intervention are supported as an option for weight loss within these guidelines, provided they are backed by evidence of their safety and efficacy.

Given the increasing prevalence of DM2 and prediabetes, their relationship to weight status, and the related health benefits of weight loss, it is important to evaluate the effectiveness of weight-loss programs for these populations in real-world settings. The purpose of this study was to evaluate the relative effectiveness of a commercial weight-loss program for weight loss in individuals with and without DM2 or high blood sugar (D/HBS). In this chart review study, we examined weight loss, body composition and several biomarkers of cardiometabolic risk over 24 weeks, in a cohort of individuals with self-reported D/HBS compared with a cohort of individuals without these conditions, all of whom were overweight/obese and enrolled in a weight-loss program featuring MRs used in conjunction with one-on-one behavioral support.

## Subjects and methods

This study is an analysis of data previously collected from three systematic retrospective chart review studies that included charts from 22 MWCC (Medifast Weight Control Centers) located in Maryland, Texas, Florida and Pennsylvania. These studies were approved by the Western Institutional Review Board (Puyallup, WA, USA), which concluded the studies met the requirements for a waiver from the informed consent process per 45 CFR 66.116(d). The study designs and data collection procedures have been published previously;^28, 29, 30^ these studies were registered in ClinicalTrials.gov (#NCT01662830 and #NCT02150837). In brief, charts from clients who were overweight or obese, and who were following one of three Medifast weight-loss meal plans (the 5 & 1 Plan, the 4 & 2 & 1 Plan, or the 5 & 2 & 2 Plan), and who had signed a personal health information consent form (which included permission to use their data for research purposes) were included in these studies. Each meal plan utilized a combination of Medifast MRs (Medifast, Inc., Owings Mills, MD, USA) and conventional foods. Each MR contained 90–110 calories, 11–15 g protein, 12–15 g carbohydrates and 0–3.5 g fat. The meal plans provide between 800–1500 calories and incorporate 4–5 MRs, 1–2 self-prepared 'lean and green meals' (each including 5–7 oz. of lean protein, three servings (~1½ cups) of non-starchy vegetables, and up to two healthy fat servings) and 0–2 healthy snacks (fruit, dairy or whole grains), depending on the plan. More details of each Medifast meal plan can be found in previous publications.^28, 29, 30^ The specific weight-loss meal plan assigned to each client was based on a number of factors including weight status, personal preferences, lifestyle, exercise habits and medical history.

The weight-loss programs included weekly one-on-one in-person sessions with trained counselors who utilized motivational interviewing to promote long-term weight control. Clients’ weight-loss goals were determined jointly by the counselor and client, which in turn determined the prescribed length of the client’s active weight-loss phase. Body weight, pulse and blood pressure were abstracted at baseline and up to 24 weeks, plus at the final visit (FV). The FV was defined as the client’s last visit to the MWCC during active weight loss while following their original meal plan; the time of the FV varied by individual client, and includes clients who stopped their assigned meal plan early. Abdominal circumference, body composition (measured by bioelectric impedance) and self-reported medical history including baseline use of prescription medication(s) were also abstracted from the charts.

### Statistical analysis

This study was a retrospective cohort analysis by self-reported diabetes status (that is, self-reported history of D/HBS versus not reported (control)). The primary outcome measure for this analysis was change in body weight (absolute and %) from baseline to weeks 4, 12 and 24.

Data were analyzed according to a pre-defined statistical analysis plan. Non-parametric Mann–Whitney *U-*tests were used for between group comparisons to examine change from baseline to weeks 4, 12, 24 as well as FV. Non-parametric paired Wilcoxon signed rank tests were used for within group comparisons at these time points.

For the primary analysis, a completers analysis was used; this analysis included each chart that had data for the given outcome and time point, irrespective of whether the individual completed his/her entire program. Similar analyses were conducted on secondary outcomes. An intention-to-treat last observation carried forward (ITT LOCF) analysis, pre-specified in the analysis plan, was also performed for the primary outcome for comparison. If missing, imputed data were carried through from the last measured observation to each client’s last prescribed week of weight loss. The ITT LOCF analyses included all charts with baseline weight data and for which the prescribed program length did not exceed the specified time point. In addition, a mixed effects regression model was used to examine weight change over time in the combined sample. Weight was the dependent variable, D/HBS vs control was the categorical predictor and baseline weight, meal plan, time and gender were covariates. The proportions of individuals achieving ⩾5 and ⩾10% weight loss from baseline were calculated, and *χ*^2^-tests were used to determine if proportions were significantly different between groups. Significance was defined as *P*<0.05. Analyses were conducted using SPSS Version 14.0 and Stata Version 10.

## Results

### Baseline characteristics

All charts from the three previous MWCC chart review studies^28, 29, 30^ that had self-reported medical history (*n*=816 of 818) were included in this analysis. The cohort with D/HBS had a higher proportion of males, weighed more, had more individuals with a BMI in the higher obesity classes, and was older than the control cohort (Table 1). Those with D/HBS were assigned to the 4 & 2 & 1 (1100–1300 kcal per day) and 5 & 2 & 2 (1300–1500 kcal per day) meal plans more often than the control cohort, which had a higher proportion using the 5 & 1 Plan (800–1000 kcal per day). The cohort with D/HBS had higher amounts of fat and lean mass compared with the control cohort, although body fat percentages were not different (Table 1). Of the 125 individuals who self-reported a history of D/HBS, 79 (63%) reported the use of prescription medication(s) for DM2 at baseline (not shown). Continued use or changes in DM2 medication was not routinely tracked in the charts.

### Program information

The mean prescribed program length of the cohort with D/HBS was ~3 weeks longer than that of the control cohort (*P*<0.0001), reflecting their higher baseline weight (Table 1). Similarly, the cohort with D/HBS stayed on their weight-loss meal plan ~3 weeks longer compared with controls (*P*=0.041).

### Body weight

**Figure 1:**
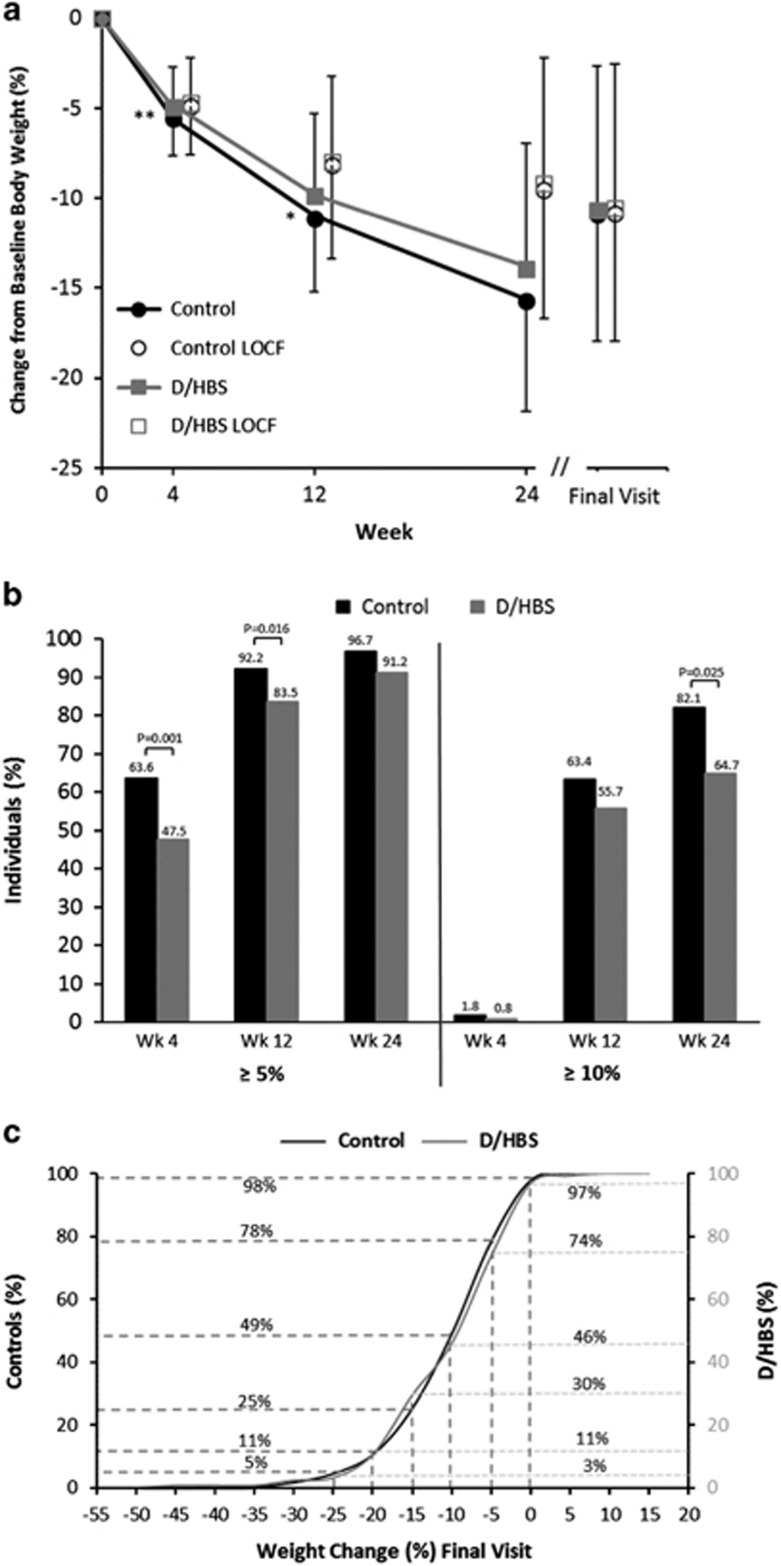
(**a**) Percent change from baseline body weight. Mean (±s.d.) for the completers population, which included all individuals with weight data at the given time point. Intention-to-Treat Last Observation Carried Forward (LOCF) values are also shown for each time point. Final visit represents an individual’s last visit to the weight control center while on their originally prescribed meal plan. Sample sizes at each time point are shown in Table 2. Between group comparison using non-parametric Mann–Whitney *U*-test: **P=0.005, *P=0.027. (**b**) Proportion of individuals with at least 5% and at least 10% reduction in baseline body weight. Analysis of the completers population, which included all individuals with weight data at the given time point; sample sizes at each time point are shown in Table 2. *χ*^2^-tests were used to determine whether proportions were significantly different between cohorts. (**c**) Cumulative percentage of individuals with at least X% change in body weight at their final visit. Final visit represents an individual’s last visit to the weight control center while on their originally assigned meal plan.

Overall, mean body weight in both cohorts decreased steadily and significantly compared with baseline through 24 weeks and at FV (Table 2 and Figure 1a). The completers analyses showed similar amounts of absolute weight lost in both cohorts at all time points (Table 2). As a percentage of baseline weight (Figure 1a), controls lost more weight than those with D/HBS at early time points (5.6±2.1 vs 5.0±2.3%, *P*=0.005 at week 4; 11.1±4.1 vs 9.9±4.6%, *P*=0.027 at week 12), whereas there were no differences between the cohorts at week 24 (15.7±6.1 vs 13.9±7.0%, *P*=0.151) or FV (10.9±7.1 vs 10.7±8.0%, *P*=0.550) in the completers analysis. Similar trends in absolute weight loss were observed in the ITT LOCF analyses, with no significant differences between cohorts (Table 2). There were no differences between cohorts in percent weight loss at any time points in the ITT LOCF analysis (Figure 1a). To account for differences in baseline characteristics between the two cohorts, weight loss was also examined in a mixed model regression analysis with adjustments for baseline weight, gender and meal plan. Both adjusted and unadjusted values are shown in Table 2. Weight-loss values in these analyses were similar to the completers analyses, and there were no differences in the amount of body weight lost between the cohorts with or without adjustments for these covariates at any of the time points or FV.

Both cohorts had significant proportions that achieved a therapeutic level of weight loss (⩾5%) (Figure 1b). More individuals in the control cohort achieved this milestone at earlier time points (weeks 4 and 12) compared with the cohort with D/HBS. However, over 90% of individuals in both cohorts lost ⩾5% of their baseline weight by week 24, with no difference between the cohorts. At 12 weeks, a majority of individuals (and similar proportions) in both cohorts experienced weight losses of ⩾10%. At week 24, there was a higher proportion of controls (82.1%) with ⩾10% weight loss compared with the cohort with D/HBS (64.7%, *P*=0.025).

Figure 1c shows the proportion of each cohort that achieved various degrees of weight loss by FV. Nearly all individuals in both cohorts lost weight by FV: ~98% in the control cohort and 97% in those with D/HBS. In addition, nearly identical proportions from both the control cohort and the cohort with D/HBS achieved weight losses of ⩾5% (78 vs 74%, respectively), ⩾10% (49 vs 46%, respectively) and ⩾20% (11% for both cohorts) by FV. A measurable proportion of both cohorts also had weight losses of ⩾25% (5% in controls and 3% in those with D/HBS).

Approximately two-thirds of the cohort with D/HBS reported using medications for DM2 at baseline. To examine potential effects of medication use on weight loss, the cohort with D/HBS was divided into a subgroup that reported use of medications for DM2 at baseline and a subgroup that did not. A completers analysis showed no difference in absolute or percent weight lost between these subgroups at any time points (data not shown).

### Body composition

**Figure 2:**
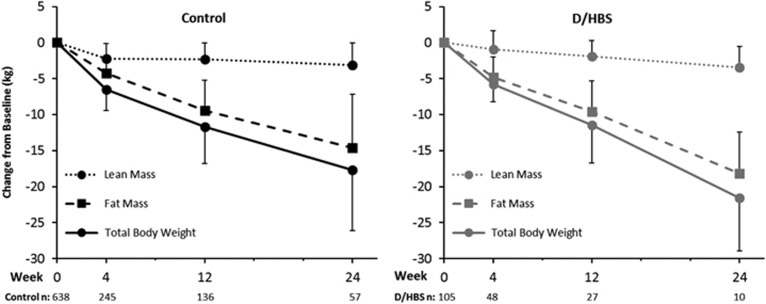
Change from baseline body weight, lean body mass and body fat mass. Mean (±s.d.) for the completers population, which included all individuals with body composition data at the given time point; sample sizes are designated below the graphs.

Weight loss in both cohorts was primarily due to decreases in fat mass (Figure 2). Fat mass represented 67, 80 and 82% of the total weight lost for controls and 87, 89 and 86% of the total weight lost in the cohort with D/HBS at 4, 12 and 24 weeks, respectively. At 4 weeks, the control cohort had lost more lean mass than those with D/HBS (2.2±2.1 vs 0.9±2.6 kg, *P*=0.002), but by 12 weeks there were no longer differences between the cohorts in the absolute amount of either lean or fat mass lost. Both cohorts lost approximately 5–6% of their baseline lean mass across the 24-week time period, whereas absolute fat mass was reduced by 32% (controls) and 40% (D/HBS) from baseline (data not shown). This resulted in positive changes in overall body composition in both cohorts: lean mass as a percentage of total body weight increased from 55–56% at baseline to 63–65% at 24 weeks, and fat mass as a percentage of total body weight decreased from 44–45 to 36–37% at 24 weeks (data not shown).

### Cardiometabolic risk factors

The mean baseline blood pressure in both cohorts was above normal (Table 3). A reduction in both systolic and diastolic blood pressure occurred early in weight loss in both cohorts, with the majority of the response occurring within the first 4 weeks (Table 3). The magnitude of the reductions in systolic and diastolic blood pressure were not different between the two cohorts, and both represented significant improvements within each cohort (*P*<0.0001). Pulse decreased at all time points throughout the study for both cohorts with no differences between the cohorts. Abdominal circumference significantly decreased from baseline at all time points throughout the study; at week 24 the control cohort had a greater reduction compared with the cohort with D/HBS (15.7±6.7 vs 10.1±6.8 cm, *P*=0.027) (Table 3).

## Discussion

Weight loss is one of the primary recommendations made to individuals with newly diagnosed DM2 to reduce complications and better manage the disease; for those with prediabetes, weight loss is recommended to prevent progression to DM2.^31^ The Look AHEAD study demonstrated that people with DM2 using an intensive lifestyle intervention can lose therapeutic amounts of weight.^22^ However, physiological as well as psychological barriers to weight loss may be more pronounced in people with DM2 or prediabetes.^11^ Few studies have directly compared the effectiveness of a weight-loss program in people with and without DM2 and, when they have done so, the results have varied.^9, 11, 14, 15, 16, 17, 18^ In this study we sought to determine whether there were differences in weight loss between individuals with self-reported DM2/HBS compared with those without when both cohorts were using structured reduced calorie meal plans and receiving behavioral support. When controlling for baseline differences, we found that weight losses were similar between cohorts (Table 2). Although there were some modest differences in the proportion who achieved 5% and 10% weight loss at some time points, there were no differences at FV. Nearly two-thirds of the cohort with D/HBS had lost ⩾5% of their body weight by the FV while on their original weight-loss plan (including people who stopped the program early); this amount of weight loss is associated with improvements in glucose control and cardiometabolic risk factors.^8, 9^ In addition, nearly half had lost ⩾10% of their baseline body weight by FV, a percentage that has been associated with reduced cardiovascular events and death in DM2.^10^ Current obesity treatment guidelines recommend 5–10% weight loss over a 6-month period.^27^ For those in the cohort with D/HBS who continued the program for 24 weeks, 91% had lost ⩾5% and 65% had lost ⩾10% of their baseline body weight. The weight loss observed in those with D/HBS at 24 weeks was 16.3 kg (13.9%) (11.4 kg; 9.2% in the ITT analysis). Based on a systematic review and meta-analysis, most studies in individuals with DM2 utilizing diet with and without physical activity and/or behavioral interventions report weight losses in the range of 2.5–6 kg and <6% at 6 months.^9^

The weight-loss intervention in this study utilized structured, reduced calorie meal plans using MRs combined with individualized one-on-one weekly counseling to create a comprehensive lifestyle program. Previous studies have shown these meal plans to be safe and effective for weight loss in individuals who were overweight or obese—including older adults (⩾ 65 years), males and females, and the morbidly obese.^28, 29, 30, 32, 33^ One previous study showed that the use of similar MRs specially formulated for DM2 was more effective for weight loss and weight maintenance than a standard, self-selected diet based on recommendations by the American Diabetes Association in people with DM2 in a clinical setting.^20^ The present study extends these findings and shows the current MR products and programs administered through a commercial weight loss center are similarly effective for weight loss in adults with and without D/HBS. It is notable that of the interventions evaluated in a systematic review and meta-analysis examining weight loss in DM2, those utilizing MRs were the only interventions to average >5% weight loss at 6 months.^9^ To further highlight the potential benefit of MRs, the 6-month weight loss for individuals with diabetes and prediabetes in a medically supervised MR program were similar to the weight loss observed in this study for the cohort with D/HBS.^14^

Retention of lean mass during weight loss is important for long-term weight maintenance and also for maintaining physical function after weight loss. The amount of fat lost on an absolute basis, and as a proportion of the total weight lost, was similar between the two cohorts, suggesting there was not a difference in the way these cohorts utilized adipose tissue to meet the caloric deficit in their diets. This is in contrast to other research which found that individuals with DM2 lost less fat per unit of body weight lost than those without DM2.^15^ Indeed, the only body composition differences in this study occurred at week 4, where the cohort with D/HBS lost less lean mass compared with controls and had a greater percentage of weight loss from fat. At later time points the losses in fat mass were similar, with >80% of weight loss due to loss of fat mass in both cohorts. Thus both cohorts had good retention of lean mass (6% or less absolute loss of lean mass across the study period). Although modest exercise was encouraged during the weight-loss program, there was no formal exercise component. The nutritional composition of these meal plans, which provide a relatively high level of protein (~100–150 g per day), is likely an important contributing factor in the retention of lean mass.^34^

Because individuals with DM2 are at a higher risk for cardiovascular disease, weight loss-related improvements in cardiometabolic risk factors are an important outcome measure. Clinically relevant and similar improvements in both blood pressure (systolic and diastolic) and pulse were observed in both cohorts. The reduction in both systolic and diastolic blood pressure and pulse occurred early in weight loss, with the majority of the response occurring within the first 4 weeks, corresponding to the time interval when individuals lost an average of 5% of baseline body weight. The magnitude of the blood pressure reductions observed in this study (10–11 mm Hg systolic and 4–6 mm Hg diastolic) has been associated with a substantial reduction in cardiovascular disease risk.^35^

Central obesity is associated with increased risk of developing DM2 and its comorbidities and has been shown to independently and adversely impact mortality.^36, 37^ Each 5 cm increment in waist circumference above 90 cm for men and 70 cm for women is associated with a 7–9% increase in mortality,^37^ and as such, we observed clinically relevant improvements in abdominal circumference in both cohorts, ranging from ~5cm decrease at 4 weeks to 10–15 cm decreases at 12 and 24 weeks. In contrast to previous research,^15^ abdominal circumference appeared to decrease more in controls at 24 weeks, although relatively few individuals remained in the cohort with D/HBS at this time point. Plausible explanations include a higher proportion of males and/or the presence of insulin resistance in the cohort with D/HBS, which may have impacted the change in central adiposity. Alternatively, changes in abdominal circumference may be directly related to an individual’s percent weight loss, and in this sample, a higher proportion of controls achieved ⩾10% weight loss at week 24 compared with the cohort with D/HBS (82.1 vs 64.7%). This abdominal circumference finding is interesting and points to an area of future research comparing long-term abdominal fat loss in individuals with and without DM2.

Laboratory outcomes such as fasting glucose, HbA1c, plasma insulin and blood lipids were not available, however, based on existing research and the percentage weight loss achieved, these laboratory values would be expected to improve;^8, 9, 10^ further prospective research would be needed to confirm this. Although some clients reported reduced DM2 medication usage on an *ad hoc* basis, suggesting improved glycemic control, comprehensive data capturing changes in medication were not available from the charts. In evaluating weight loss for those who reported DM2 medication use at baseline versus those who did not, we found no differences in absolute or percent weight loss.

Given the data were retrospective and medical history was self-reported, there are limitations to describe. Related to the cohort assignment, the presence of D/HBS was not verified by laboratory measure or physician records; data were not available to definitively differentiate between D/HBS, so it could not be determined directly what proportions had prediabetes, were newly diagnosed with DM2, or had a diagnosis of longer duration. However, two-thirds of those in the cohort with D/HBS reported using DM2 medications at baseline, so it is reasonable to assume that this proportion represented individuals with DM2 rather than prediabetes. In addition, individual medications were not uniformly documented so the impact of specific diabetes therapies on weight could not be evaluated. It is also possible, given the number of undiagnosed individuals with these conditions, that some individuals included in the control cohort may have had undiagnosed prediabetes or DM2. Despite this possibility, self-reported diabetes diagnosis has been shown to be reliable in several studies evaluating various populations.^38, 39, 40^ Laboratory outcomes related to glycemic control and lipids were also not available. The retrospective cohort design resulted in differences in the baseline characteristics of the controls and those with D/HBS. These baseline differences reflect typical differences between populations with and without DM2 or prediabetes.^14^ Despite these differences in baseline characteristics, similar amounts of weight (absolute, percentage) were lost, and after correcting for baseline weight, gender and meal plan assignment, there was no difference in the rate of weight loss between the cohorts. Moreover, given the large proportion of those with D/HBS reporting use of DM2 medications, and that individuals using DM2 medications may be more resistant to weight loss,^9^ overall weight loss for the cohort with D/HBS did not appear to be negatively impacted. Both of these points suggest the weight-loss program was similarly effective in adults with DM2 or prediabetes, and in those without these conditions.

In contrast to most prospective weight loss studies that limit the study population to generally healthy individuals with obesity, a strength of this study was the broad selection criteria, which included many individuals with baseline health issues in addition to DM2 and prediabetes.^29, 30^ The high number of centers represented, the relatively large sample size and the broad chart selection criteria support the generalizability of the study results among weight control center customers and provide a true picture of real-life experience on this program.

### Conclusion

This study reinforces previous findings that structured weight-loss programs incorporating MRs and behavioral support can be an effective approach for weight loss in adults who are overweight and obese, including those with DM2 or prediabetes. In this study examining a real-world setting, adults with self-reported D/HBS experienced similar weight loss and fat mass losses, as well as improvements in measured cardiometabolic risk factors, as a cohort without these conditions.

## Figures and Tables

**Table 1:** Baseline characteristics

*Characteristic*	*Control*	*Diabetes/high blood sugar*	P*-value*
*n*	691	125	
Gender *n* (%)			<0.0001
Female	514 (74.4)	74 (59.2)	
Male	177 (25.6)	51 (40.8)	
Age years (s.d.)	48.2 (12.6)	56.3 (11.1)	<0.0001
Weight kg (s.d.) (*n*=815)	102.8 (25.7)	112.6 (25.6)	<0.0001
BMI kg m^−^^2^ (s.d.) (*n*=812)	36.27 (7.64)	38.96 (8.13)	<0.0001
			
*BMI class* n *(%)*			
Overweight	130 (18.9)	13 (10.5)	0.048
Class 1	218 (31.7)	34 (27.4)	
Class 2	158 (23.0)	31 (25.0)	
Class 3	181 (26.3)	46 (37.1)	
			
*Body composition (*n=*743)*			
Fat mass % (s.d.)	43.7 (7.5)	44.0 (7.6)	0.791
Fat mass kg (s.d.)	45.2 (15.1)	48.6 (15.7)	0.014
Lean mass kg (s.d.)	56.8 (14.2)	60.0 (12.6)	0.003
Systolic blood pressure mm Hg (s.d.) (*n*=650)	127.7 (17.2)	131.8 (17.3)	0.034
Diastolic blood pressure mm Hg (s.d.) (*n*=650)	85.7 (11.0)	83.7 (9.8)	0.145
Pulse BPM (s.d.) (*n*=645)	75.6 (11.9)	77.2 (12.6)	0.292
Abdominal circumference cm (s.d.) (*n*=734)	120.8 (17.5)	126.4 (18.6)	0.002
			
*Meal plan* n *(%)*			
5 & 1	400 (57.9)	44 (35.2)	<0.0001
4 & 2 & 1	245 (35.5)	65 (52.0)	
5 & 2 & 2	46 (6.7)	16 (12.8)	
Prescribed program weeks (s.d.) (*n*=813)	22.7 (14.1)	26.0 (12.4)	<0.0001
Actual program weeks (s.d.) (*n*=804)	18.0 (13.7)	20.8 (15.2)	0.041

Abbreviations: BMI, body mass index; BPM, beats per minute.

**Table 2:** Absolute change in body weight

	*Weight in kg—mean (s.d.)*											
	*4 weeks*			*12 weeks*			*24 weeks*			*Final Visit*		
	n	*Baseline*	*Change*	n	*Baseline*	*Change*	n	*Baseline*	*Change*	n	*Baseline*	*Change*
*Completers*												
Control^a^	601	103.1 (25.4)	−5.8 (2.5)	383	104.3 (24.8)	−11.6 (5.1)	151	109.0 (26.3)	−17.1 (7.9)	682	102.9 (25.7)	−11.3 (8.3)
D/HBS^a^	118	113.1 (25.5)	−5.6 (2.7)	79	113.7 (24.0)	−11.0 (5.2)	34	118.7 (21.8)	−16.3 (8.6)	122	112.4 (24.9)	−11.9 (9.7)
*P*-value^b^			0.697			0.423			0.590			0.431
												
*ITT LOCF*												
Control^a^	688	102.8 (25.7)	−5.0 (3.1)	559	107.6 (25.4)	−8.7 (6.0)	243	124.8 (25.5)	−11.8 (9.0)	690^c^	102.8 (25.7)	−11.2 (8.3)
D/HBS^a^	124	112.6 (25.7)	−5.3 (2.9)	114	115.5 (24.6)	−9.0 (5.4)	66	125.8 (24.4)	−11.4 (8.5)	125^d^	112.6 (25.6)	−11.8 (9.7)
*P*-value^b^			0.343			0.497			0.884			0.479
												
*Mixed model regression*												
Control^e,f^	690	103.1 [102.8] (2.1)	−5.7 [−5.7] (2.5)	690	103.5 [102.8] (4.2)	−11.5 [−11.5] (5.0)	690	104.1 [102.8] (5.2)	−16.9 [−16.8] (7.7)	681^g^	101.2 [103.0] (8.2)	−11.3 [−11.2] (8.3)
D/HBS^e,f^	125	112.9 [112.6] (1.9)	−5.6 [−5.6] (2.7)	125	113.0 [112.6] (3.6)	−11.0 [−10.9] (5.2)	125	113.8 [112.6] (4.6)	−16.2 [−15.9] (8.6)	122^h^	111.5 [112.6] (7.1)	−11.9 [−12.0] (9.7)
*P*-value^i^			0.589 [0.578]			0.279 [0.338]			0.287 [0.604]			0.420 [0.394]

Abbreviations: D/HBS, DM2 or high blood sugar; ITT LOCF, intent-to-treat last observation carried forward. Between group comparisons were assessed using non-parametric Mann–Whitney *U*-test and within group comparisons were assessed using a non-parametric paired Wilcoxon signed rank test comparing repeated measures in a single sample.

a Within group changes from baseline were significant at all time points (*P*<0.0001).

b Between group comparisons.

c Two controls did not have data for prescribed number of weeks and therefore were included only in final visit analysis.

d One D/HBS did not have data for prescribed number of weeks and therefore was included only in final visit analysis.

e Mean adjusted for baseline weight, gender and meal plan [unadjusted mean] (s.d. for adjusted).

f Within group change from baseline significant at all time points (*P*<0.05).

g [unadjusted *n*=690].

h [unadjusted *n*=125].

i Between group difference adjusted [unadjusted].

**Table 3:** Change in cardiometabolic risk factors

	*Mean (s.d.)*											
	*4 Weeks*			*12 weeks*			*24 weeks*			*Final visit*		
	n	*Baseline*	*Change*	n	*Baseline*	*Change*	n	*Baseline*	*Change*	n	*Baseline*	*Change*
*SBP (mm Hg)*												
Control^a^	475	127.7 (17.0)	−8.2 (16.2)	299	126.8 (16.1)	−9.3 (16.2)	117	130.8 (18.2)	−11.4 (17.6)	503	128.2 (17.4)	−9.1 (16.5)
D/HBS^b^	71	132.0 (17.4)	−11.6 (15.3)	45	131.6 (15.2)	−13.2 (18.2)	20	134.2 (17.8)	−19.6 (21.9)	66	131.1 (16.6)	−11.2 (18.6)
*P*-value^c^			0.121			0.198			0.215			0.385
												
*DBP (mmHg)*												
Control^a^	475	85.7 (11.1)	−5.2 (10.8)	299	85.2 (11.0)	−6.0 (11.2)	117	86.4 (11.9)	−6.7 (12.3)	504	86.0 (11.1)	−5.7 (11.3)
D/HBS^d^	71	83.9 (9.4)	−6.7 (10.2)	45	84.6 (10.1)	−8.1 (9.2)	20	85.1 (9.5)	−8.5 (12.1)	66	83.6 (10.1)	−6.2 (10.3)
*P*-value^c^			0.467			0.262			0.721			0.766
												
*Pulse (BPM)*												
Control^d^	471	75.1 (11.6)	−2.8 (10.3)	296	74.4 (11.4)	−3.2 (11.0)	116	73.9 (10.6)	−2.9 (9.8)	501	75.6 (11.8)	−2.9 (11.8)
D/HBS^e^	71	76.5 (12.8)	−3.6 (10.8)	44	77.2 (13.9)	−7.3 (13.1)	20	78.1 (13.1)	−6.8 (13.1)	65	77.8 (12.5)	−4.3 (11.9)
*P*-value^c^			0.310			0.061			0.474			0.481
												
*AC (cm)*												
Control^a^	283	122.8 (17.0)	−5.4 (4.2)	154	121.2 (16.3)	−10.8 (6.0)	59	124.0 (15.2)	−15.7 (6.7)			
D/HBS^f^	55	126.8 (19.9)	−4.9 (6.3)	33	122.0 (11.1)	−9.6 (5.3)	8	126.1 (11.2)	−10.1 (6.8)			
*P*-value^c^			0.108			0.265			0.027			

Abbreviations: AC, abdominal circumference; BPM, beats per minute; DBP, diastolic blood pressure; D/HBS, DM2 or high blood sugar; SBP, systolic blood pressure. Between group comparisons were assessed using non-parametric Mann–Whitney *U*-test and within group comparisons were assessed using a non-parametric paired Wilcoxon signed rank test comparing repeated measures in a single sample.

a Within group changes from baseline were significant at all time points (*P*<0.0001).

b Within group changes from baseline were significant at all time points (*P*⩽0.002).

c Between group comparisons.

d Within group changes from baseline were significant at all time points (*P*⩽0.007).

e Within group changes from baseline were significant at weeks 4, 12 and FV (*P*<0.01).

f Within group changes from baseline were significant at all time points (*P*⩽0.012).

## References

[bib1] International Diabetes Federation. IDF diabetes atlas seventh edition. International diabetes federation; 2014.

[bib2] Vos T, Barber RM, Bell B, Bertozzi-Villa A, Biryukov S, Bolliger I et al. Global, regional, and national incidence, prevalence, and years lived with disability for 301 acute and chronic diseases and injuries in 188 countries, 1990-2013: a systematic analysis for the Global Burden of Disease Study 2013. Lancet 2015; 386: 743–800. 26063472 10.1016/S0140-6736(15)60692-4 PMC4561509

[bib3] Centers for Disease Control and Prevention. National diabetes statistics report: estimates of diabetes and its burden in the united states, 2014. Services USDoHaH: Atlanta, GA, USA, 2014.

[bib4] Stokes A, Preston SH. Deaths attributable to diabetes in the United States: comparison of data sources and estimation approaches. PLoS One 2017; 12: e0170219. 28121997 10.1371/journal.pone.0170219 PMC5266275

[bib5] Boyle JP, Thompson TJ, Gregg EW, Barker LE, Williamson DF. Projection of the year 2050 burden of diabetes in the US adult population: dynamic modeling of incidence, mortality, and prediabetes prevalence. Popul Health Metr 2010; 8: 29. 20969750 10.1186/1478-7954-8-29 PMC2984379

[bib6] Knowler WC, Barrett-Connor E, Fowler SE, Hamman RF, Lachin JM, Walker EA et al. Reduction in the incidence of type 2 diabetes with lifestyle intervention or metformin. N Engl J Med 2002; 346: 393–403. 11832527 10.1056/NEJMoa012512 PMC1370926

[bib7] Garvey WT, Ryan DH, Henry R, Bohannon NJ, Toplak H, Schwiers M et al. Prevention of type 2 diabetes in subjects with prediabetes and metabolic syndrome treated with phentermine and topiramate extended release. Diabetes Care 2014; 37: 912–921. 24103901 10.2337/dc13-1518 PMC4392900

[bib8] Wing RR, Lang W, Wadden TA, Safford M, Knowler WC, Bertoni AG et al. Benefits of modest weight loss in improving cardiovascular risk factors in overweight and obese individuals with type 2 diabetes. Diabetes Care 2011; 34: 1481–1486. 21593294 10.2337/dc10-2415 PMC3120182

[bib9] Franz MJ, Boucher JL, Rutten-Ramos S, VanWormer JJ. Lifestyle weight-loss intervention outcomes in overweight and obese adults with type 2 diabetes: a systematic review and meta-analysis of randomized clinical trials. J Acad Nutr Diet 2015; 115: 1447–1463. 25935570 10.1016/j.jand.2015.02.031

[bib10] Look AHEAD Research Group, Gregg EW, Jakicic JM, Blackburn G, Bloomquist P, Bray GA et al. Association of the magnitude of weight loss and changes in physical fitness with long-term cardiovascular disease outcomes in overweight or obese people with type 2 diabetes: a post-hoc analysis of the Look AHEAD randomised clinical trial. Lancet Diabetes Endocrinol 2016; 4: 913–921. 27595918 10.1016/S2213-8587(16)30162-0 PMC5094846

[bib11] Pi-Sunyer FX. Weight loss in type 2 diabetic patients. Diabetes Care 2005; 28: 1526–1527. 15920086 10.2337/diacare.28.6.1526

[bib12] Van Gaal L, Scheen A. Weight management in type 2 diabetes: current and emerging approaches to treatment. Diabetes Care 2015; 38: 1161–1172. 25998297 10.2337/dc14-1630

[bib13] Franz MJ. The dilemma of weight loss in diabetes. Diabetes Spectr 2007; 20: 133–136.

[bib14] Li Z, Tseng CH, Li Q, Deng ML, Wang M, Heber D. Clinical efficacy of a medically supervised outpatient high-protein, low-calorie diet program is equivalent in prediabetic, diabetic and normoglycemic obese patients. Nutr Diabetes 2014; 4: e105. 24513578 10.1038/nutd.2014.1 PMC3940825

[bib15] Baker ST, Jerums G, Prendergast LA, Panagiotopoulos S, Strauss BJ, Proietto J. Less fat reduction per unit weight loss in type 2 diabetic compared with nondiabetic obese individuals completing a very-low-calorie diet program. Metabolism 2012; 61: 873–882. 22146094 10.1016/j.metabol.2011.10.017

[bib16] Wing RR, Marcus MD, Epstein LH, Salata R. Type II diabetic subjects lose less weight than their overweight nondiabetic spouses. Diabetes Care 1987; 10: 563–566. 3677974 10.2337/diacare.10.5.563

[bib17] Campos GM, Rabl C, Mulligan K, Posselt A, Rogers SJ, Westphalen AC et al. Factors associated with weight loss after gastric bypass. Arch Surg 2008; 143: 877–883; discussion 884. 18794426 10.1001/archsurg.143.9.877 PMC2747804

[bib18] Deng ML, Wang M, Tseng C-H, Li Z, Heber D. Rates of weight loss in diabetics versus non-diabetics in subjects in an outpatient very low calorie diet program. FASEB J 2012; 26: 826.

[bib19] Heymsfield SB, van Mierlo CA, van der Knaap HC, Heo M, Frier HI. Weight management using a meal replacement strategy: meta and pooling analysis from six studies. Int J Obes Relat Metab Disord 2003; 27: 537–549. 12704397 10.1038/sj.ijo.0802258

[bib20] Cheskin LJ, Mitchell AM, Jhaveri AD, Mitola AH, Davis LM, Lewis RA et al. Efficacy of meal replacements versus a standard food-based diet for weight loss in type 2 diabetes: a controlled clinical trial. Diabetes Educ 2008; 34: 118–127. 18267998 10.1177/0145721707312463

[bib21] Heymsfield SB. Meal replacements and energy balance. Physiol Behav 2010; 100: 90–94. 20193699 10.1016/j.physbeh.2010.02.010

[bib22] Look ARG, Pi-Sunyer X, Blackburn G, Brancati FL, Bray GA, Bright R et al. Reduction in weight and cardiovascular disease risk factors in individuals with type 2 diabetes: one-year results of the look AHEAD trial. Diabetes Care 2007; 30: 1374–1383. 17363746 10.2337/dc07-0048 PMC2665929

[bib23] Franz MJ, VanWormer JJ, Crain AL, Boucher JL, Histon T, Caplan W et al. Weight-loss outcomes: a systematic review and meta-analysis of weight-loss clinical trials with a minimum 1-year follow-up. J Am Diet Assoc 2007; 107: 1755–1767. 17904936 10.1016/j.jada.2007.07.017

[bib24] Harrigan M, Cartmel B, Loftfield E, Sanft T, Chagpar AB, Zhou Y et al. Randomized trial comparing telephone versus in-person weight loss counseling on body composition and circulating biomarkers in women treated for breast cancer: the Lifestyle, Exercise, and Nutrition (LEAN) Study. J Clin Oncol 2016; 34: 669–676. 26598750 10.1200/JCO.2015.61.6375 PMC4872022

[bib25] Appel LJ, Clark JM, Yeh HC, Wang NY, Coughlin JW, Daumit G et al. Comparative effectiveness of weight-loss interventions in clinical practice. N Engl J Med 2011; 365: 1959–1968. 22085317 10.1056/NEJMoa1108660 PMC4074540

[bib26] Wadden TA, West DS, Neiberg RH, Wing RR, Ryan DH, Johnson KC et al. One-year weight losses in the Look AHEAD study: factors associated with success. Obesity (Silver Spring) 2009; 17: 713–722. 19180071 10.1038/oby.2008.637 PMC2690396

[bib27] Jensen MD, Ryan DH, Apovian CM, Ard JD, Comuzzie AG, Donato KA et al. AHA/ACC/TOS guideline for the management of overweight and obesity in adults: a report of the American College of Cardiology/American Heart Association Task Force on practice guidelines and the Obesity Society. Circulation 2014; 129: S102–S138. 24222017 10.1161/01.cir.0000437739.71477.ee PMC5819889

[bib28] Coleman C, Kiel J, Hanlon-Mitola A, Sonzone C, Fuller N, Davis LM. Use of the Medifast meal replacement program for weight loss in overweight and obese clients: a retrospective chart review of three Medifast Weight Control Centers (MWCC). Food Nutr Sci 2012; 03: 1433–1444.

[bib29] Coleman CD, Kiel JR, Mitola AH, Langford JS, Davis KN, Arterburn LM. Effectiveness of a Medifast meal replacement program on weight, body composition and cardiometabolic risk factors in overweight and obese adults: a multicenter systematic retrospective chart review study. Nutr J 2015; 14: 77. 26245279 10.1186/s12937-015-0062-8 PMC4527127

[bib30] Kiel JR, Coleman CD, Mitola AH, Langford JS, Davis KN, Arterburn LM. The effectiveness of a partial meal replacement program in extremely obese individuals: a systematic retrospective chart review of Medifast Weight Control Centers. J Obes Weight Loss Ther 2015; S5: 007. 10.1186/s12937-015-0062-8 PMC4527127 26245279

[bib31] American Diabetes Association. Obesity management for the treatment of type 2 diabetes. Sec. 7. In Standards of Medical Care in Diabetes-2017. Diabetes Care 2017; 40: S57–S63. 27979894 10.2337/dc17-S010

[bib32] Davis LM, Coleman C, Kiel J, Rampolla J, Hutchisen T, Ford L et al. Efficacy of a meal replacement diet plan compared to a food-based diet plan after a period of weight loss and weight maintenance: a randomized controlled trial. Nutr J 2010; 9: 11. 20222968 10.1186/1475-2891-9-11 PMC2851659

[bib33] Shikany JM, Thomas AS, Beasley TM, Lewis CE, Allison DB. Randomized controlled trial of the Medifast 5 & 1 Plan for weight loss. Int J Obes (Lond) 2013; 37: 1571–1578. 23567927 10.1038/ijo.2013.43 PMC3836833

[bib34] Pasiakos SM, Margolis LM, Orr JS. Optimized dietary strategies to protect skeletal muscle mass during periods of unavoidable energy deficit. FASEB J 2015; 29: 1136–1142. 25550460 10.1096/fj.14-266890

[bib35] McInnes GT. Lowering blood pressure for cardiovascular risk reduction. J Hypertens Suppl 2005; 23: S3–S8. 10.1097/01.hjh.0000165622.34192.fd 15821449

[bib36] Tchernof A, Despres JP. Pathophysiology of human visceral obesity: an update. Physiol Rev 2013; 93: 359–404. 23303913 10.1152/physrev.00033.2011

[bib37] Cerhan JR, Moore SC, Jacobs EJ, Kitahara CM, Rosenberg PS, Adami HO et al. A pooled analysis of waist circumference and mortality in 650,000 adults. Mayo Clin Proc 2014; 89: 335–345. 24582192 10.1016/j.mayocp.2013.11.011 PMC4104704

[bib38] Schneider AL, Pankow JS, Heiss G, Selvin E. Validity and reliability of self-reported diabetes in the atherosclerosis risk in communities study. Am J Epidemiol 2012; 176: 738–743. 23013620 10.1093/aje/kws156 PMC3571247

[bib39] Yuan X, Liu T, Wu L, Zou ZY, Li C. Validity of self-reported diabetes among middle-aged and older Chinese adults: the China Health and Retirement Longitudinal Study. BMJ Open 2015; 5: e006633. 10.1136/bmjopen-2014-006633 PMC4401856 25872937

[bib40] Sheikh MA, Lund E, Braaten T. Test-retest reliability of self-reported diabetes diagnosis in the Norwegian Women and Cancer Study: A population-based longitudinal study (*n* =33919). SAGE Open Med 2016; 4: 2050312115622857. 26835013 10.1177/2050312115622857 PMC4724769

